# ABEILLE: a novel method for ABerrant Expression Identification empLoying machine LEarning from RNA-sequencing data

**DOI:** 10.1093/bioinformatics/btac603

**Published:** 2022-09-05

**Authors:** Justine Labory, Gwendal Le Bideau, David Pratella, Jean-Elisée Yao, Samira Ait-El-Mkadem Saadi, Sylvie Bannwarth, Loubna El-Hami, Véronique Paquis-Fluckinger, Silvia Bottini

**Affiliations:** Université Côte d’Azur, Center of Modeling, Simulation and Interactions, Nice 06000, France; Université Côte d’Azur, Inserm U1081, CNRS UMR 7284, Institute for Research on Cancer and Aging, Nice (IRCAN), Centre Hospitalier Universitaire (CHU) de Nice, Nice 06200, France; Université Côte d’Azur, Inserm U1081, CNRS UMR 7284, Institute for Research on Cancer and Aging, Nice (IRCAN), Centre Hospitalier Universitaire (CHU) de Nice, Nice 06200, France; Université Côte d’Azur, Center of Modeling, Simulation and Interactions, Nice 06000, France; Université Côte d’Azur, Center of Modeling, Simulation and Interactions, Nice 06000, France; Université Côte d’Azur, Inserm U1081, CNRS UMR 7284, Institute for Research on Cancer and Aging, Nice (IRCAN), Centre Hospitalier Universitaire (CHU) de Nice, Nice 06200, France; Université Côte d’Azur, Inserm U1081, CNRS UMR 7284, Institute for Research on Cancer and Aging, Nice (IRCAN), Centre Hospitalier Universitaire (CHU) de Nice, Nice 06200, France; Université Côte d’Azur, Center of Modeling, Simulation and Interactions, Nice 06000, France; Université Côte d’Azur, Inserm U1081, CNRS UMR 7284, Institute for Research on Cancer and Aging, Nice (IRCAN), Centre Hospitalier Universitaire (CHU) de Nice, Nice 06200, France; Université Côte d’Azur, Inserm U1081, CNRS UMR 7284, Institute for Research on Cancer and Aging, Nice (IRCAN), Centre Hospitalier Universitaire (CHU) de Nice, Nice 06200, France; Université Côte d’Azur, Center of Modeling, Simulation and Interactions, Nice 06000, France

## Abstract

**Motivation:**

Current advances in omics technologies are paving the diagnosis of rare diseases proposing a complementary assay to identify the responsible gene. The use of transcriptomic data to identify aberrant gene expression (AGE) has demonstrated to yield potential pathogenic events. However, popular approaches for AGE identification are limited by the use of statistical tests that imply the choice of arbitrary cut-off for significance assessment and the availability of several replicates not always possible in clinical contexts.

**Results:**

Hence, we developed ABerrant Expression Identification empLoying machine LEarning from sequencing data (ABEILLE) a variational autoencoder (VAE)-based method for the identification of AGEs from the analysis of RNA-seq data without the need for replicates or a control group. ABEILLE combines the use of a VAE, able to model any data without specific assumptions on their distribution, and a decision tree to classify genes as AGE or non-AGE. An anomaly score is associated with each gene in order to stratify AGE by the severity of aberration. We tested ABEILLE on a semi-synthetic and an experimental dataset demonstrating the importance of the flexibility of the VAE configuration to identify potential pathogenic candidates.

**Availability and implementation:**

ABEILLE source code is freely available at: https://github.com/UCA-MSI/ABEILLE.

**Supplementary information:**

Supplementary data are available at *Bioinformatics* online.

## 1 Introduction

Omics technologies have revolutionized the world of biology and medicine tailoring the way to next-generation healthcare. The advent of whole-exome sequencing (WES) and whole-genome sequencing (WGS) has greatly accelerated the identification of variants in previously unknown rare disease genes (Wortmann *et al.*, 2015). Although these technologies are mainstays in Mendelian disease diagnosis, their success rate for detecting causal variants is far from complete, ranging from 25 to 50% (Taylor *et al.*, 2015). Several variants remain as variants of unknown significance (VUS) or they are missed due to the inability to prioritize them. Recently the employ of RNA sequencing (RNA-seq) has been proposed (Byron *et al.*, 2016). This technology provides a direct probing of RNA abundance and sequence of both coding and non-coding genome, including allele-specific expression and splice isoforms (Wang *et al.*, 2009). Despite the very promising premises of RNA-seq to detect new variants, the pioneering works using this technique improved the diagnostic power by only 10% (Cummings *et al.*, 2017; Frésard *et al.*, 2019; Gonorazky *et al.*, 2019; Kremer *et al.*, 2017; Lee *et al.*, 2020). Transcriptome analysis facilitates genome-wide interpretation of DNA variants, specifically three aberrant events can be analyzed: aberrant expression, aberrant splicing, allelic imbalance, or allele-specific expression. Currently, some improvements have been achieved in the identification of aberrant splicing events and allele-specific expression thanks to the development of novel tools designed for rare diseases such as FRASER (Mertes *et al.*, 2021), LeafCutterMD (Jenkinson *et al.*, 2020) and ANEVA-DOT (Mohammadi *et al.*, 2019). On the other hand, the identification of aberrant gene expression (AGE) in this context requires a paradigm shift toward a novel way to analyze gene expression data. Usually, to identify AGE, a comparison of gene expression levels between two groups of individuals with different conditions such as healthy/diseased, exposed/unexposed to treatment, or others, is carried out. The significance of the results is measured by statistical tests whose statistical power decreases if the two groups are made up of too few individuals (Khang and Lau, 2015). This approach is not adapted in the context of rare diseases. By definition, rare diseases concern only a very small number of subjects, thus the availability of large cohorts is uncommon. Most importantly, very often, replicates for the same individual are not available in a such clinical context. Furthermore, the disposal of a control group, such as healthy individuals, is often limited. Moreover, the heterogeneity of these pathologies is very high, which means that the same disease present in several patients will not be due to the same responsible genes. Consequently, these individuals cannot be combined to constitute the diseased group. Finally, Li *et al.*, they showed that most of the popular methods for detecting differentially expressed genes, identify an elevated number of false positives (Li *et al.*, 2022). Therefore, classical statistical methods cannot be used for the detection of AGEs in the context of rare diseases. Two approaches have been proposed to fulfill these needs. OUTRIDER (Brechtmann *et al.*, 2018), which combines an autoencoder and a statistical test to identify AGE, and OutPyR (Salkovic *et al.*, 2020) which uses a Bayesian model. Although OUTRIDER showed good results on the Kremer *et al.* dataset, its performance relies on considerable sample size (more than 60 samples) not always possible in the context of rare diseases. OutPyR showed good performances in reporting injected AGE on small sample size but the number of total AGE identified was very high leading to a high number of false positives. Consequently, there is an urgent burning to develop novel computational approaches to resolve the diagnostic deadlock and improve our knowledge of rare disorders (Labory *et al.*, 2020; Rahman and Rahman, 2018). Here, we describe ABEILLE, (ABerrant Expression Identification empLoying machine LEarning from sequencing data) a VAE-based method for the identification of AGE from RNA-seq data without the need for replicates and without assumption on the distribution, using a flexible model obtained after testing several parameters. We compare its performances to the state-of-the-art alternatives, OUTRIDER (Brechtmann *et al.*, 2018) and OutPyR (Salkovic *et al.*, 2020), using semi-synthetic data and a real dataset.

## 2 Materials and methods

### 2.1 Datasets

**Rare-disease cohort from Kremer *et al***. The RNA-seq raw read counts for this cohort were downloaded from Supplementary Data S1 (Kremer *et al.*, 2017). The cohort is composed of 119 patients with suspected mitochondrial disease in diagnostic stalemate for which RNA has been extracted from fibroblasts and sequenced.

**Small datasets**. We used the Kremer *et al.* dataset and we created six smaller sub-datasets of size 110, 90, 60, 30, 20, and 10 samples, respectively, composed of the 6 patients with validated AGE (MUC1344, MUC1365, MUC1396, MUC1404, MUC1350, and MUC1361) and the complementary number of patients to arrive to the desired dataset size. For each size, we created 10 datasets randomly selecting the patients without the validated AGE. We then run the two tools ABEILLE and OUTRIDER and we measured how many times they were able to identify any combination of the six AGEs depending on the size of the dataset.

**GTEx cohort**. The Genotype-Tissue Expression (GTEx) project (Consortium, 2015) is a public database of WGS, WES, and RNA-seq data collected *post-mortem* from 54 non-diseased tissue sites across nearly 1000 individuals. We randomly selected samples from the GTEx database to build the semi-synthetics datasets. We also filtered out samples with RIN < 6 (RNA Integrity Number).

**Semi-synthetic datasets**. Due to the absence of a gold standard real dataset with known AGE, we decided to use a computational strategy to inject AGE in the GTEx cohort. We implemented the same strategy as described in the study of Brechtmann *et al.* (2018). Briefly, raw read counts in the original dataset were replaced by:

(1) $$k_{ij}^{O}=\mathrm{round}(s_{i}2^{\mu_{j}^{u}\pm \exp\left(N\right)\sigma_{j}^{u}})$$

With $k_{ij}^{O}$, the generated count for gene *j* and sample *i* to substitute to the original value, $s_{i}$ is the size number, $\mu_{j}^{u}$ the mean of u across the gene *j*, $\sigma_{j}^{u}$ the standard deviation and as described in (Brechtmann *et al.*, 2018), *N* is the amplitude of the corrupted count [a random value drawn in a normal distribution characterized by a mean of log(3) and a standard deviation of log(1.6)]. AGEs are injected randomly with probability 10^−6^, 10^−5^ or 10^−4^ resulting in different percentages of injected AGEs (0.001‰, 0.01‰ or 0.1‰ respectively) in samples randomly selected from the GTEx database. We created 18 semi-synthetic datasets composed of 50, 75, 125, 250, 500 and 1000 samples with AGE injected with the three different probabilities. For a complete summary of the semi-synthetic dataset characteristics see Supplementary Table S1.

### 2.2 Algorithm

**Fig. 1.**
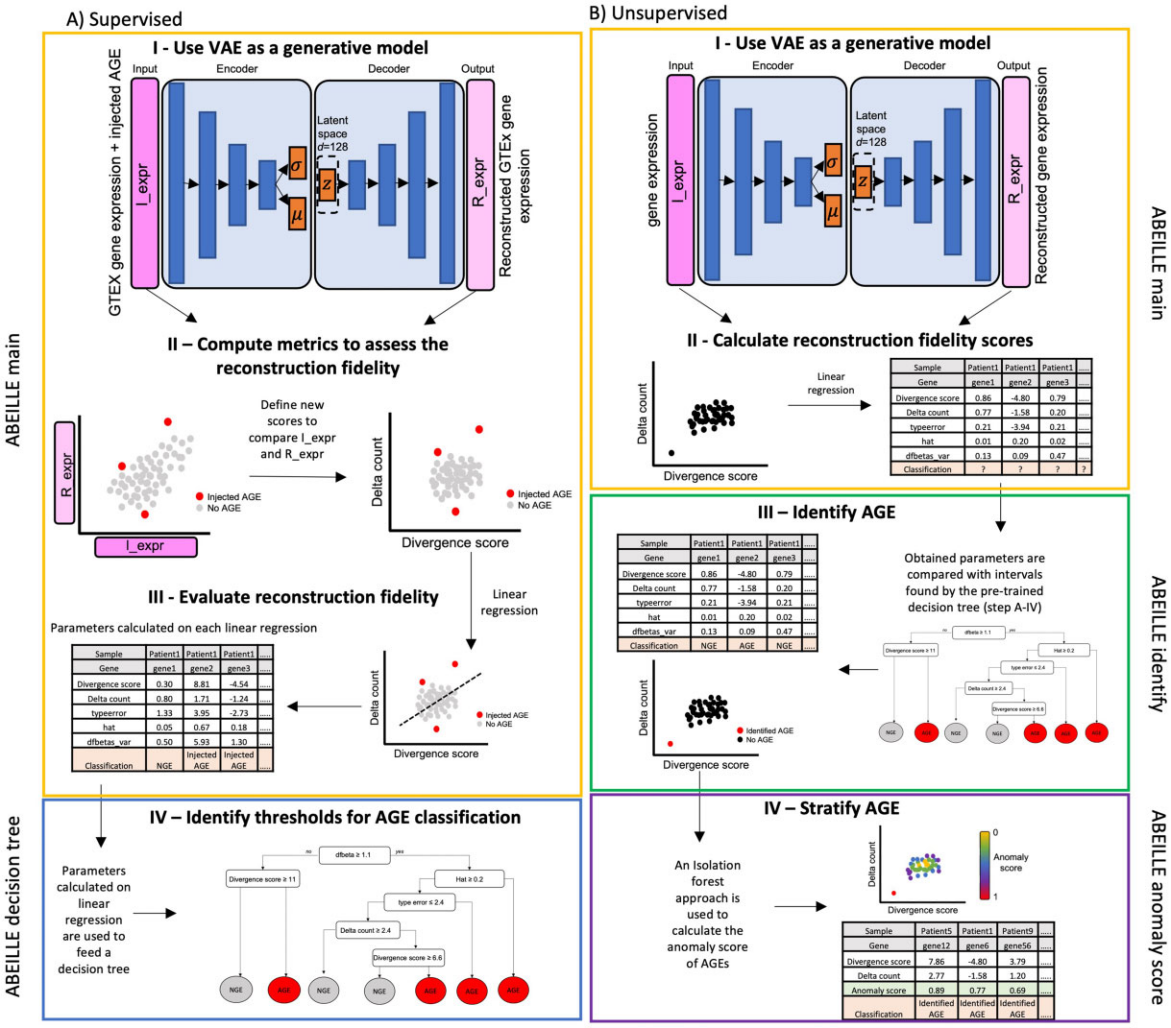
The workflow of ABEILLE to identify AGE. It is comprised of two phases: (**A**) a supervised phase to identify parameters intervals of aberration and (**B**) an unsupervised one to identify AGE. (A) We feed the VAE with the semi-synthetic dataset (I_expr) and let the model generate the reconstructed counts (R_expr) (A-I). AGEs present in the original dataset represent a perturbation to the data distribution. The integrity of reconstructed values by the VAE is compromised for AGEs. Thus, comparing I_expr and R_expr should lead the identification of AGEs (A-II left). To enhance the comparison and evaluate the reconstruction fidelity, we established two novel metrics: the divergence score and the delta count (A-II right). For each gene, the divergence score and delta count are plotted for each patient composing the cohort. We thus apply a linear regression model and calculate parameters associated to this model to evaluate its position on the plot and its reconstruction fidelity (A-III). These parameters were used to feed a decision tree using the CART algorithm. The obtained decision tree is showed in A-IV. This decision tree gives the intervals of the regression parameters to classify gene expression as AGE or NGE. (B) The first step is to run the VAE and the linear regression model on unlabeled dataset in order to calculate the regression parameters on the values of divergence score and delta count obtained by comparing I_expr and R_expr (B-I and B-II). The procedure is the same as described for the supervised phase. The obtained regression parameters on the unlabeled dataset are compared to the intervals obtained with *ABEILLE decision tree* during the supervised phase. This allow to classify gene expression as AGE or NGE based on the regression parameters (B-III). An isolation forest approach is used to calculate the anomaly score to be associated to AGEs (B-IV)

ABEILLE is a computational framework to identify AGEs (Fig. 1). It is composed of two phases: a supervised phase to identify parameters intervals of aberration and an unsupervised one to identify AGEs.

#### 2.2.1 Supervised phase

The purpose of this phase is to determine the thresholds of parameters implemented in ABEILLE model to discriminate AGEs respect to normal gene expression (NGE). For this phase, we use two modules: *ABEILLE main* and *ABEILLE decision tree.* Hence, a labeled dataset with known AGEs must be used. In absence of that, as it is the case for us, a semi-synthetic dataset can be built. AGEs can be simulated as described in Brechtmann *et al.* (2018) and described in the paragraph ‘Datasets’.

**ABEILLE main**. The core of ABEILLE is composed by a VAE followed by a linear regression model.

VAEs are generative models, which means they learn to approximate a data generating distribution. Through approximation and compression, these models have been shown to capture an underlying data manifold (a constrained, lower-dimensional space where data is distributed) and disentangle sources of variation from different classes of data.

The variational auto-encoder was built with Tensorflow (Martín Abadi *et al.*, 2015). The VAE is composed of the input and the output, four hidden layers in the encoder and in the decoder, and the special latent space of a VAE. The sampling process and the lost function of the VAE is from the Kingma *et al.* study (Kingma and Welling, 2014). Each layer of the encoder or decoder is composed of the dense layer followed by a batch normalization and the ELU activation function. More details on the VAE configuration are in Supplementary Material.

We feed the VAE with the semi-synthetic dataset (I_expr) and let the model generate the reconstructed counts (R_expr) (Fig. 1A-I). AGEs present in the original dataset represent a perturbation to the data distribution. The integrity of reconstructed values by the VAE is compromised for AGEs. Thus, comparing I_expr and R_expr should lead the identification of AGEs (Fig. 1A-II left). To enhance the comparison and evaluate the reconstruction fidelity, we established two novel metrics: the divergence score and the delta count (Fig. 1A-II right). The divergence score *D*. It measures the divergence between the reconstructed values for each gene by the VAE and the original values. We define the divergence score as:

(2) $${\;D}_{ij}=\frac{L_{ij}-\mu_{i}^{L}}{\sigma_{i}^{L}},$$

Where $L_{ij}=\left(\frac{k_{ij}+1}{{\hat{k}}_{ij}+1}\right),k_{ij}$ is the original raw count for gene *j* and sample *i*, ${\hat{k}}_{ij}$ is the reconstructed count for gene *j* and sample *i*, $\mu_{i}^{L}$ is the mean of $L_{ij}$ across all the patients and where $\sigma_{i}^{L}$ is the standard of deviation of $L_{ij}$ across all the patients.

The delta count Δ, that we define here as:

(3) $${\;\Delta }_{ij}=\log_{2}\left(\frac{{\hat{k}}_{ij}}{\mu_{j}^{k}}\right)$$

where ${\hat{k}}_{ij}$ is the reconstructed count for gene *j* and sample *i*, $\mu_{j}^{k}\;$is the mean of the original counts for gene *j*.

For each gene, the divergence score and delta count are plotted for each patient composing the cohort: we expect the points to be agglomerated very close to each other in case of NGE (high reconstruction fidelity) while to see point(s) further from the main agglomeration in case of AGE (low reconstruction fidelity). We thus apply a linear regression model and calculate parameters associated to this model to evaluate its position on the plot and its reconstruction fidelity. These parameters are:

- *Typeerror*, the standard residues is the distance between the predicted value on the linear regression and the real value.
- *Dfbetas*, are statistics that indicate the effect that deleting each one observation has on the estimates of the regression coefficients associated with the gene.
- *Hat*, projection matrix where the value of the diagonal corresponds to leverages. The leverages describe the influence that each response value has on the fitted value for that same observation.
- *CooksD*, Cook’s distance is defined as the sum of all the changes in the regression model when observation i is removed from it.

**ABEILLE decision tree.** To avoid to set threshold for the parameters calculated on the linear regression to define the AGE, we used a decision tree approach. Regression parameters calculated on the semi-synthetic datasets were used to feed a decision tree using the CART algorithm (Fig. 1A-III). The obtained decision tree is showed in Figure 1A-IV. This decision tree gives the intervals of the regression parameters to classify gene expression as AGE or NGE. Once calculated, these intervals can be used on unlabeled datasets to classify gene expression by comparing reconstructed counts by the VAE with the input gene counts through the linear regression model.

#### 2.2.2 Unsupervised phase

This phase can be run once obtained the aberration intervals through the supervised phase. Alternatively, the aberration intervals found in this study can be used without re-running the supervised phase. The unsupervised phase allows to classify gene expression on unlabeled datasets.

**ABEILLE main**. The first step is to run the VAE and the linear regression model on unlabeled dataset in order to calculate the regression parameters on the values of divergence score and delta count obtained by comparing I_expr and R_expr (Fig. 1B-I and II). The procedure is the same as described for the supervised phase.

**ABEILLE identify**. The decision tree calculated in the supervised phase lead us the intervals of regression parameters to classify gene expression as AGE or NGE. During this step, the regression parameters calculated by *ABEILLE main* on the unlabeled dataset are compared with the intervals obtained with *ABEILLE decision tree*. This allows to classify gene expression as AGE or NGE based on the regression parameters.

**ABEILLE anomaly score**. Since ABEILLE is not a statistical method, classical calculation of *P*-value is not applicable. Thus, to score the predicted AGE, we employed an isolation forest approach. Just like the random forests, isolation forests are built using decision trees. They are implemented in an unsupervised fashion as there are no pre-defined labels. Isolation forests were designed with the idea that anomalies are ‘few and distinct’ data points in a dataset. Each observation, i.e. a pair of divergence score—delta count, is given an anomaly score. The closer the score is to 1 the higher is the anomaly. We run this approach on each plot of divergence score and delta count for each gene (Fig. 1B-IV) and we retrieve the anomaly scores for predicted AGE from *ABEILLE identify*.

### 2.3 Benchmark of AGE detection methods

We compared the performances of ABEILLE, OUTRIDER and OutPyR. OUTRIDER and OutPyR were used with their respective default parameters. We calculated precision, recall and F1 score on semi-synthetic data by counting the number of AGE injected and identified and the AGE injected but not identified with the total number of AGE identified or AGE injected. Precision-recall curves were built by ranking identified AGE by *P*-value or Z-score for OUTRIDER and by divergence score or delta count for ABEILLE.

### 2.4 Functional analysis

To identify enriched terms in AGEs found in the Kremer dataset by ABEILLE, OUTRIDER and the case-control approach used in the original study, we used the R package enrichR (Kuleshov *et al.*, 2016). EnrichR interrogates 10 gene-set libraries and finds enriched terms using a hypergeometric test. Results are reported only for significant enrichment (*P*-value < 0.05).

### 2.5 Exploring ABEILLE VAE features

We developed a Shiny app to allow exploration of ABEILLE encoded feature dimensions with covariate information. The app is available at https://jlabory.shinyapps.io/ABEILLE-main/.

### 2.6 Implementation

ABEILLE is implemented in Python and R. The use of the VAE allowing the reconstruction of the data can be done in two different ways, either using the script abeille.py in a standalone way, or by importing the python function through the framework in R, the totality of the remaining steps working in R. Once the data is reconstructed, it is enough to import them in R and then to compute the parameters of divergence score and delta count. The IdentifyAGE function will then retrieve these parameters as well as the original data and the reconstructed data in order to select the AGEs.

OUTRIDER is an R package available on Bioconductor. It can be used in two different ways: either by using the OUTRIDER function which allows to launch the whole analysis or by proceeding step by step. To start the analyses, you must first filter out the unexpressed genes using the filterExpression function. The next step is to fit the count data to a negative binomial distribution, and then calculate the *P*-values. Finally, the last step of the pipeline consists in calculating the Z-scores.

OutPyR is fully implemented in Python. It uses a Bayesian model to identify AGEs. OutPyR takes as input an expression matrix in raw counts. Unlike ABEILLE and OUTRIDER, there is no filtering step to remove low expressed genes. OutPyR computes a *P*-value for each couple of gene-patient and returns a matrix of *P*-values. We retained AGEs if the *P*-values is less than or equal to 0.05.

## 3 Results

### 3.1 ABEILLE VAE features capture biological signals

**Fig. 2.**
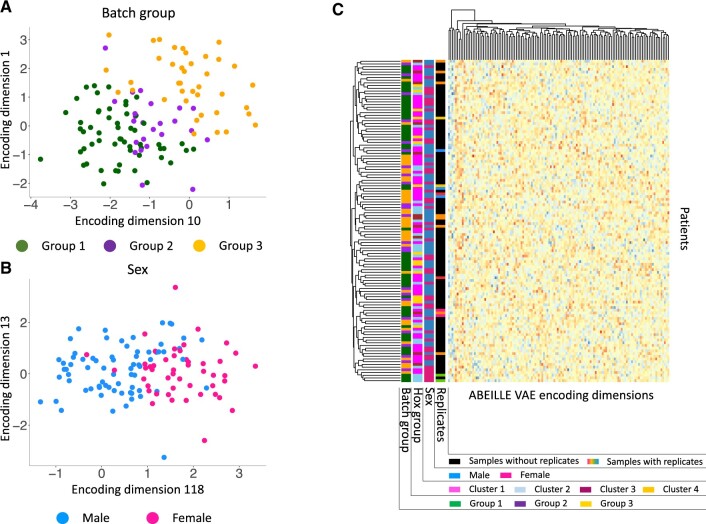
ABEILLE VAE features captures biological signals. (**A**) Encoding dimensions 10 and 1 stratify patients batch group. (**B**) Encoding dimension 118 separates patients by sex. (**C**) Full ABEILLE encoding dimensions by Kremer sample heatmap. Patients on the *y* axis, biological features on the *x* axis according to the legend in the plot

First, we tested the ABEILLE VAE on the Kremer dataset. The ABEILLE VAE compressed the patients into a lower-dimensional space, the latent space, composed by 128 features dimension. The goal was to evaluate ABEILLE on its ability to learn biological signals in the data. Since ABEILLE VAE works in unsupervised fashion, the compressed features dimension encoded in the latent space can represent known or unknown biological patterns. Therefore, we investigated whether or not ABEILLE could distinguish the biological characteristics available for this cohort: the patient sex and the batch group. Feature dimension 118 nearly perfectly separates samples by sex (Fig. 2A) and feature dimension 110 by batch group (Fig. 2B) indicating that ABEILLE VAE model patient sex and bath group robustly. We then explored all the 128 features dimension of the latent space (Fig. 2C). The heatmap shows that features are non-redundant and highly heterogeneous. Based on the hierarchical clustering dendrogram, we can see that these features capture distinct signals: for instance, hox group are large signals present in these data (Kremer *et al.*, 2017) but they are uniformly distributed in the dendrogram indicating non-redundant features activations. Overall, these observations showed the ability of the ABEILLE VAE to model gene expression data of patients through the identification of features dimensions that encode for biological patterns.

### 3.2 Divergence score, delta count and anomaly score: three novel metrics to identify AGE

VAE are well-established generative models with the ability to reconstruct original data with high fidelity (Kingma and Welling, 2014). Since AGE represents a perturbation to the gene raw count distribution, the integrity of the reconstruction is compromised for AGEs. Thus, the comparison of the original and reconstructed counts allows the identification of AGEs. The divergence score and delta count are two novel metrics introduced here to evaluate the reconstruction fidelity of the original gene expression counts by the VAE model. Once AGEs are identified, they are scored by an isolation tree approach that allows to calculate the anomaly score: the closer this score is to 1, the more severe is the aberration.

**Fig. 3.**
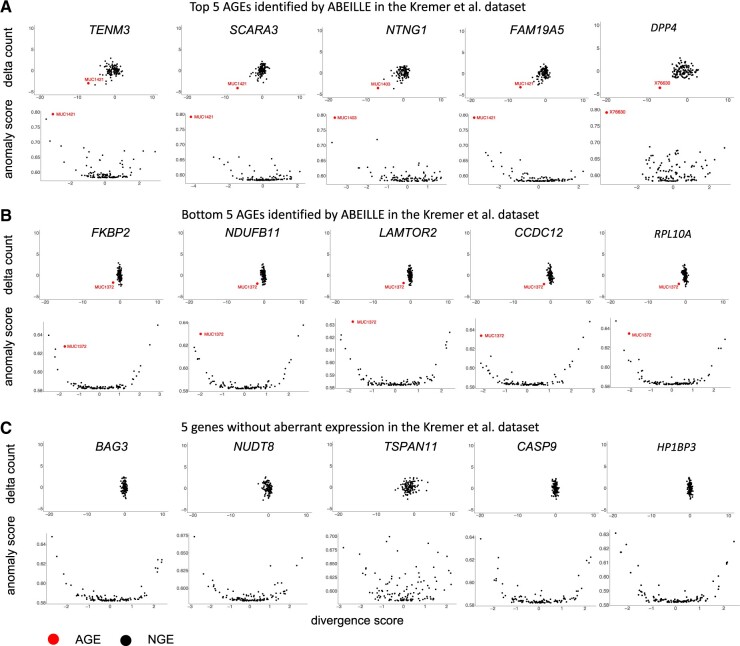
The three novel parameters defined in ABEILLE: the divergence score, the delta count and the Aberrant Score. Measure of the divergence score versus delta count (first row) and divergence score versus anomaly score (second row) (**A**) representing the top five AGEs and (**B**) the bottom five AGEs found by ABEILLE in the Kremer dataset sorted by anomaly score; (**C**) five randomly selected genes where no aberrant expression was found in any patient of the cohort. The red point represents the AGE with the patient identifier indicated closer to the point and black point the NGE

We tested these three scores on the Kremer dataset. After using ABEILLE to identify AGEs, we plotted the divergence score, delta count and anomaly score for the top and the bottom five AGEs sorted by anomaly score as well as five randomly chosen NGE (Fig. 3). We can observe that the divergence score and delta count allow to easily identify AGE as the point far from the main points distribution. Similarly, by comparing the distance of the AGE from the rest of the distribution of the top five compared with the bottom five, we can see the importance of the anomaly score to sort the aberration.

Then we focused on the experimentally validated pathogenic candidates in the original publication. These genes have different sources of aberration in specific patients: MGST1 was identified by AGE in patient MUC1396, TIMMDC1 was identified by AGE and aberrant splicing in two patients MUC1344 and MUC1365, MCOLN1 in patient MUC1361 was identified by AGE although with a signal slightly inferior of the thresholds of significance for *Z*-score and *P*-values set by the authors, ALDH18A1 was identified by aberrant splicing with AGE at the limit of thresholds for patient MUC1404, finally in patient MUC1350, CLPP was identified by allele specific-expression only without any consequences on the expression level. ABEILLE correctly identified as AGE the genes MGST1 and TIMMDC1 but could not identify ALDH18A1 (Supplementary Fig. S1). However, this gene mainly shows aberrant splicing, that is not calculated by ABEILLE. The gene MCOLN1, as well as in the original publication, can be retrieved as AGE if thresh-olds are relaxed allowing to include this gene. Finally, CLPP was not identified as AGE as expected, because, as well as in the original study, this gene was not identified as AGE but only having allele specific-expression. Overall, ABEILLE could retrieve the AGE for the pathogenic candidates identified by Kremer with exception of ALDH18A1.

Altogether these analyses demonstrated that the divergence score, delta count and anomaly score are valid parameters to identify AGE.

### 3.3 AGE detection on a real dataset

We studied the performance of the three tools, ABEILLE, OUTRIDER and OutPyR to detect AGEs on real data from Kremer *et al.* (2017) and we compared with the classical case-control analysis performed with DESeq2 by the original study. We found that OutPyR identified a number of AGEs considerably higher than the other approaches (Supplementary Table S2).

**Fig. 4.**
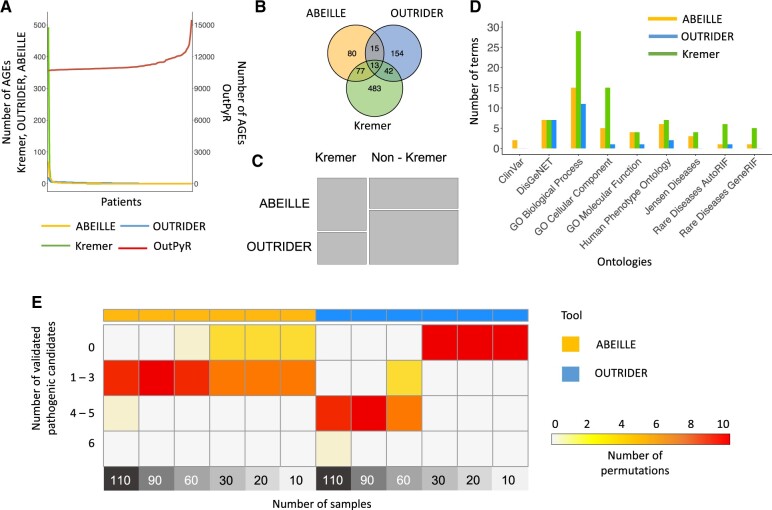
Benchmark of ABEILLE, OUTRIDER and OutPyR on real data. (**A**) The number of AGEs per patient is reported sorted in descending order for ABEILLE, OUTRIDER and Kremer, in ascending order for OutPyR. (**B**) Venn diagram representing the number of AGEs shared by ABEILLE, OUTRIDER and Kremer. (**C**) Mosaic plot showing the proportion of AGEs shared by ABEILLE and Kremer or OUTRIDER and Kremer with respect to not shared AGEs. (**D**) Summary of the functional analysis results performed on AGEs identified by the 3 approaches on 11 ontologies regarding terms related to mitochondrial biology and diseases. (**E**) Performances of ABEILLE and OUTRIDER on small datasets

Then we calculated the number of AGE per patient (Fig. 4A) and we can observe that the minimal number of AGE per patient identified by OutPyR is 10696. OutPyR violates the definition of AGE in rare diseases where we expect very few AGEs for each patient. These observations rule out OutPyR as a tool for AGE identification in this context.

We compared the number of AGEs found by OUTRIDER and ABEILLE with the AGE detected in the original study (Fig. 4B, Supplementary Table S3). ABEILLE identified a higher proportion of AGEs in common with Kremer *et al.* respect to OUTRIDER (*P*-value 5.289e-07, fisher test; Fig. 4C): 48% of AGE detected by ABEILLE are in common with the original study, while only 19% for OUTRIDER. Very few AGEs identified are common to ABEILLE and OUTRIDER, meaning that the two tools target different AGE populations. Consequently, when we study the intersection of the AGE found by the two tools with those identified in the original publication, we observe little overlap.

We noticed that in the original Kremer study, the number of AGE identified for one patient, MUC1372, is considerably high: 493 out of 615 for the entire cohort. Interestingly, ABEILLE found 70 AGEs for this patient, that is, the maximal number of AGE per patient reported by this tool, while OUTRIDER did not find any AGE for the same patient. Although further information about the patient and the data processing would be necessary, our hypothesis is that OUTRIDER autoencoder is too stringent thus all patterns in the data are canceled, on the other hand the case-control approach overestimates the number of AGEs by comparing each patient with all other of the cohort. In conclusion, ABEILLE seems to offer the best balance of cofounder controlling without suppression of hidden biological patterns in the data. This observation highlights the limitation of the case-control approach in this context.

Finally, we investigated the biological meaning of AGE found by ABEILLE and OUTRIDER compared to the AGE found by the case-control approach of the original study (Fig. 4D and Supplementary Fig. S2). Overall terms related to mitochondrial biology or diseases are enriched of AGE identified by ABEILLE, similarly to the AGEs identified in the original study. On the contrary, AGE identified by OUTRIDER are poorly enriched in mitochondrial-related gene-sets. Notably, a novel set of AGEs identified only by ABEILLE showed significant enrichment in ClinVar variants (Landrum *et al.*, 2014) related to mitochondrial diseases.

### 3.4 AGE detection on small dataset size

One of the main limitations in the field of rare diseases is the samples availability. Thus having a data analysis tool that do not require a consistent number of samples is pivotal. To test the performances of ABEILLE and OUTRIDER regarding this fundamental issue, we created small sub datasets of the Kremer dataset (see section 2 for more details). Briefly, we randomly suppressed samples from the original cohort in order to obtain datasets with sizes: 110, 90, 60, 30, 20 and 10 always keeping the 6 patients with validated AGEs. We repeated the strategy 10 times for each size thus obtaining 60 datasets. We then tested the ability of ABEILLE and OUTRIDER to recall any of the 6 validated AGEs accordingly with the sample size (Fig. 4E). As expected, OUTRIDER was not able to identify any validated AGE when the sample size is smaller than 60. On the contrary, ABEILLE performances are stable regarding the size of the dataset being able to identify validated AGEs also for datasets composed with less than 30 samples. This analysis demonstrates that the performances of OUTRIDER strongly depends on the number of samples provided due to the use of a statistical approach to identify AGEs, while our novel strategy independent of any statistical test, shows good performances for very small datasets. This result is relevant for the rare disease context since small cohorts are usually available.

### 3.5 Precision–recall benchmark for AGE detection

We then studied the identification of AGEs by ABEILLE and OUTRIDER. To perform this analysis, we injected simulated AGEs into the GTEx data and counted the number of such AGEs that were recovered as described in Section 2. We simulated AGEs with three different probabilities resulting in different percentages of injected AGEs (0.1‰, 0.01‰ or 0.001‰). The analysis was repeated for six different size datasets: 50, 75, 125, 250, 500, 1000 samples (Supplementary Table S1). We calculated precision, recall and F1 score to measure ABEILLE and OUTRIDER performances on the 18 semi-synthetic datasets. Regarding precision, both tools show very similar performances on all the datasets despite the size and the proportion of injected AGEs. We can assess that both tools correctly recall similar proportions of injected AGEs. Interestingly, we can observe a stratification of sensitivity of both tools accordingly with the size of the dataset. Overall, ABEILLE shows higher sensitivity than OUTRIDER especially for small datasets composed of 50 or 75 samples, while similar values of this score are obtained for datasets of size bigger than 250 samples. This result is particularly relevant in the context of rare diseases where usually only very small cohorts are available. On the other hand, for these aforementioned datasets, OUTRIDER achieved a slightly better F1 score. We observe that performances of OUTRIDER strongly depend on the proportion of AGEs injected in the dataset, showing better results when the proportion of injected AGEs is the highest simulated. This dependence is less important for ABEILLE. Also, this result is of particular note in the context of rare diseases since we expect to have very few AGEs for each patient.

To further explore the properties of the two tools, we calculated the precision-recall curves on two ranking for each tool, namely *P*-values and Z-scores for OUTRIDER and divergence score and delta count for ABEILLE (Supplementary Fig. S3). On datasets with 0.1‰ AGEs injected, ABEILLE ranking by delta count showed the higher performances than the ranking by divergence score, especially for datasets sizes above 250 and smaller than 50. When the percentage of injected AGEs diminish, the ranking by divergence score yielded better results for ABEILLE. OUTRIDER ranking by *P*-values are slightly better than by Z-score.

Overall depending on dataset size and percentage of injected AGEs, the performances of the tools depend on the score used to rank the AGEs. This result underlines that the ranking by one score and defined thresholds are not best suitable for AGE identification.

## 4 Discussion and conclusions

In this study, we presented ABEILLE, it combines the use of a VAE, able to model any type of data without any assumption about their distribution, and a decision tree, allowing to obtain a classification of genes as AGE or non-AGE. We compared the performances of ABEILLE with the state-of-the-art alternative OUTRIDER that uses an AE to normalize the data and then identify AGEs through a statistical test. The performance comparison of these two tools was done using two datasets: a semi-synthetic dataset and a real dataset from the study by Kremer *et al.*

Due to the lack of a ‘gold standard’ dataset with known real AGEs, we had to generate *in silico* datasets in order to compare the precision and recall of these two tools. One limitation of this analysis is that the GTEx database contains real AGEs over which we have no control and which suggests an underestimation of the performance of both tools with our analyses. Our results show that, although both tools have similar precision, ABEILLE has a higher recall than OUTRIDER especially for small datasets (<75 samples). Since recall expresses the proportion of correctly found AGEs among the total number of injected AGEs, we can state that ABEILLE performs better in identifying true AGEs. ABEILLE performs better on datasets with very few AGEs, which makes it particularly suitable for rare diseases where a single gene in a patient may be responsible for the pathology and where the available cohorts are small. These results were corroborated by the application on the real data from Kremer *et al.* dataset. Here ABEILLE showed a higher agreement with AGE found by the original study than OUTRIDER and a higher specificity to detect AGEs experimentally validated. Of note, ABEILLE outperformed OUTRIDER when sample size is smaller than 60 samples: while OUTRIDER cannot identify any of the validated AGE, ABEILLE showed similar performances as observed on the entire cohort. Thus ABEILLE is suitable for AGE identification in the context of rare diseases.

We did not compare ABEILLE with other known methods for RNA-Seq data analysis such as DESeq2 (Love *et al.*, 2014) or edgeR (Robinson *et al.*, 2010), because the estimation of AGEs is not based on the same principle. While these methods are suitable for identification of fold-change variation comparing two population, ABEILLE is conceived to identify AGE in one population. Moreover, these methods require several replicates to obtain reliable results and in general these replicates are not produced in the context of diagnostic research. However, it could be interesting to add these estimators to ABEILLE in order to improve the decision tree used to identify AGEs.

Finally, we tested ABEILLE and OUTRIDER on finding AGEs in single-tissues of the GTEx dataset (data available at https://zenodo.org/record/6395166). Both tools showed some limitations to handle these data, leading to a large number of genes with very low expression identified as AGE. For OUTRIDER this limitation was already claimed by the authors because of a possible poor fit of the negative binomial model. For ABEILLE, the issue is in the sparsity of the data because we optimized the model for small datasets where we do not expect a high degree of sparsity as in the single-tissue datasets in GTEx. Thus a novel model should be developed to handle single-tissue and multiple-tissues AGE identification, although we do not expect to have data from several tissues for the same patient in the diagnostic context.

In conclusion, we have demonstrated that ABEILLE is a valid alternative to OUTRIDER, especially for small cohorts composed by less than 60 samples, targeting a different population of AGEs more specific for aberrant expression than the population of AGEs identified by OUTRIDER. The advantages of ABEILLE are: ability to have good performances on small cohorts thanks to the VAE model that performs the learning phase on less data because of the sampling in the latent space, more flexibility due to the representation of each input point as a normal distribution in the latent space that should be able to approximate any other distribution and more resistant to noise.

In the future, we need to perform more tests in order to evaluate the ability of ABEILLE to work properly on different types of data and not only on RNA-Seq data. The ability to analyze different types of data could pave the way for the use of ABEILLE on multi-tissues and multi-omics data, including proteomic and metabolomic data.

## Data availability

The data underlying this article are available in the article, in its online Supplementary Material and at the repository https://zenodo.org/record/6395166. The app to explore the latent space is available at https://jlabory.shinyapps.io/ABEILLE-main/. ABEILLE source code is freely available at: https://github.com/UCA-MSI/ABEILLE.

## Funding

This work was supported by the French government, through the UCA JEDI Investments in the Future project managed by the National Research Agency (ANR) under reference number [ANR-15-IDEX-01].

*Conflict of Interest*: none declared.

## Supplementary Material

btac603_Supplementary_Data

## References

[btac603-B1] Abadi M. et al (2015) *TensorFlow: Large-Scale Machine Learning on Heterogeneous Systems*.

[btac603-B2] Ardlie K.G. et al The GTEx Consortium. (2015) The Genotype-Tissue expression (GTEx) pilot analysis: multitissue gene regulation in humans. Science, 348, 648–660. 25954001 10.1126/science.1262110 PMC4547484

[btac603-B3] Brechtmann F. et al (2018) OUTRIDER: a statistical method for detecting aberrantly expressed genes in RNA sequencing data. Am. J. Hum. Genet., 103, 907–917. 30503520 10.1016/j.ajhg.2018.10.025 PMC6288422

[btac603-B4] , Breiman L. et al (1984) *Classification and Regression Trees*. (1st ed.). Routledge. 10.1201/9781315139470

[btac603-B5] Byron S.A. et al (2016) Translating RNA sequencing into clinical diagnostics: opportunities and challenges. Nat. Rev. Genet., 17, 257–271. 26996076 10.1038/nrg.2016.10 PMC7097555

[btac603-B6] Cummings B.B. et al Genotype-Tissue Expression Consortium. (2017) Improving genetic diagnosis in Mendelian disease with transcriptome sequencing. Sci. Transl. Med., 9. 10.1126/scitranslmed.aal5209 PMC5548421 28424332

[btac603-B7] Frésard L. et al; Care4Rare Canada Consortium. (2019) Identification of rare-disease genes using blood transcriptome sequencing and large control cohorts. Nat. Med., 25, 911–919. 31160820 10.1038/s41591-019-0457-8 PMC6634302

[btac603-B8] Gonorazky H. et al (2019) *Expanding the Boundaries of RNA Sequencing as a Diagnostic Tool for Rare Mendelian Disease*. *Am. J. Hum. Gen.*, **104**,466–483. 10.1016/j.ajhg.2019.01.012 PMC6407525 30827497

[btac603-B9] Jenkinson G. et al (2020) LeafCutterMD: an algorithm for outlier splicing detection in rare diseases. Bioinformatics, 36, 4609–4615. 32315392 10.1093/bioinformatics/btaa259 PMC7750945

[btac603-B10] Khang T.F. , Lau C.Y. (2015) Getting the most out of RNA-seq data analysis. PeerJ, 3, e1360. 26539333 10.7717/peerj.1360 PMC4631466

[btac603-B11] Kingma D.P. , Welling M. (2014) Auto-encoding variational Bayes. arXiv:1312.6114 [cs, stat].

[btac603-B12] Kremer L.S. et al (2017) Genetic diagnosis of Mendelian disorders via RNA sequencing. Nat. Commun., 8, 15824. 28604674 10.1038/ncomms15824 PMC5499207

[btac603-B13] Kuleshov M.V. et al (2016) Enrichr: a comprehensive gene set enrichment analysis web server 2016 update. Nucleic Acids Res., 44, W90–W97. 27141961 10.1093/nar/gkw377 PMC4987924

[btac603-B14] Labory J. et al (2020) Multi-omics approaches to improve mitochondrial disease diagnosis: challenges, advances and perspectives. **Front. Mol. Sci.,* **2**, 590842.* 10.3389/fmolb.2020.590842 PMC7667268 33240932

[btac603-B15] Landrum M.J. et al (2014) ClinVar: public archive of relationships among sequence variation and human phenotype. Nucleic Acids Res., 42, D980–D985. 24234437 10.1093/nar/gkt1113 PMC3965032

[btac603-B16] Lee H. et al Undiagnosed Diseases Network. (2020) Diagnostic utility of transcriptome sequencing for rare Mendelian diseases. Genet. Med., 22, 490–499. 31607746 10.1038/s41436-019-0672-1 PMC7405636

[btac603-B17] Li Y. et al (2022) Exaggerated false positives by popular differential expression methods when analyzing human population samples. Genome Biol., 23, 79. 35292087 10.1186/s13059-022-02648-4 PMC8922736

[btac603-B18] Love M.I. et al (2014) Moderated estimation of fold change and dispersion for RNA-seq data with DESeq2. Genome Biol., 15, 550. 25516281 10.1186/s13059-014-0550-8 PMC4302049

[btac603-B19] Mertes C. et al (2021) Detection of aberrant splicing events in RNA-seq data using FRASER. Nat. Commun., 12, 529. 33483494 10.1038/s41467-020-20573-7 PMC7822922

[btac603-B20] Mohammadi P. et al (2019) Genetic regulatory variation in populations informs transcriptome analysis in rare disease. Science, 366, 351–356. 31601707 10.1126/science.aay0256 PMC6814274

[btac603-B21] Rahman J. , Rahman S. (2018) *Mitochondrial Medicine in the Omics Era*. 10.1016/S0140-6736(18)30727-X 29903433

[btac603-B22] Robinson M.D. et al (2010) edgeR: a bioconductor package for differential expression analysis of digital gene expression data. Bioinformatics, 26, 139–140. 19910308 10.1093/bioinformatics/btp616 PMC2796818

[btac603-B23] Salkovic E. et al (2020) OutPyR: Bayesian inference for RNA-Seq outlier detection. J. Comput. Sci., 47, 101245.

[btac603-B24] Taylor J.C. et al (2015) Factors influencing success of clinical genome sequencing across a broad spectrum of disorders. Nat. Genet., 47, 717–726. 25985138 10.1038/ng.3304 PMC4601524

[btac603-B25] Wang Z. et al (2009) RNA-Seq: a revolutionary tool for transcriptomics. Nat. Rev. Genet., 10, 57–63. 19015660 10.1038/nrg2484 PMC2949280

[btac603-B26] Wortmann S.B. et al (2015) Whole exome sequencing of suspected mitochondrial patients in clinical practice. J. Inherited Metab. Dis., 38, 437–443. 25735936 10.1007/s10545-015-9823-y PMC4432107

