# Supplementary material for: ABEILLE: a novel method for ABerrant Expression Identification empLoying machine LEarning from RNA-sequencing data
Source: Bioinformatics. 2022 Sep 5;38(20):4754–61. doi: 10.1093/bioinformatics/btac603 (PMC9563686; doi:10.1093/bioinformatics/btac603)
Supplement: btac603_Supplementary_Data [file btac603_supplementary_data.zip › supplementary methods.docx]

*Variational auto-encoder*

This is the core component of ABEILLE. The variational auto-encoder was built with Tensorflow (Abadi et al., 2015). The VAE is composed of the input and the output, four hidden layers in the encode and in the decoder, and the special latent space of a VAE (Figure 1A). The sampling process and the lost function of the VAE is from the Kingma et al. study (Kingma and Welling, 2013). Each layer of the encoder or decoder is composed of the dense layer followed by a batch normalization and the ELU activation function.

In order to build an autoencoder able to model any data beyond sequencing counts and without assumption on the distribution, we needed to establish a flexible model.

The first step is the definition of the likelihood function. Following Kingma et al. (Kingma and Welling, 2013), we built the loss function as:

$$L_{VAE}= -KL\left( q_{\theta}\left( z | x \right) \right| p_{\theta}(z))+\frac{1}{L} \sum_{l=1}^{L} \log p_{\theta}(x|z)$$

Where $p_{\theta}$ is the prior, in our case it is normal gaussian reparametrized, $q_{\theta}$ is the posterior (the distribution of the latent space), $KL$ is the Kullback-Leibler divergence.

And then we implement the loss function:

$$KL\left( q_{\theta}\left( z | x \right) \right| p_{\theta}(z))=\frac{1}{2}\sum(\exp\varepsilon\left( x \right)+\mu^{2}\left( x \right)-1-\varepsilon\left( x \right))$$

With $\varepsilon$ the variance of the latent space and $\mu$ its mean. For the reconstruction loss we used the mean squared log error between the output and the input of the VAE:

$$\frac{1}{L} \sum_{l=1}^{L} \log p_{\theta}(x|z)=\frac{1}{N}\sum_{i=1}^{N} {\log\left( \frac{X_{i}+1}{\hat{X_{i}}+1} \right)}^{2}$$

Here we call $X_{ij}$ the input of the VAE and $\hat{X}_{ij}$ the output. We also define $k_{ij}$ the read counts and $\hat{k}_{ij}$ the reconstruction of the read counts as output of the VAE.

In the following table we report how we tuned all parameters for VAE architecture. Tests were performed on several semi-synthetic datasets generated as explained in the methods section of the manuscript.

| Parameters | Observations |
| --- | --- |
| The number of hidden layers | Tested increasing step by step the number of hidden layers. After 4 hidden layers in each of the encoder and the decoder the model can get worse. |
| The number of neurons in each layer | The $2^{n}$ convention works fine starting the latent space at 128. |
| Batch size | The batch size is fine-tuned around 6% of the number of samples in the dataset. |
| Activation function | After testing all the classical activation function (sigmoid, tanh, mish…) the best performing one is exponential linear unit. |
| Batch normalization | Tested configuration without the batch normalization to all layers. A slight gain of accuracy for the model, but it adds the possibility to crash at the fitting part. |
| Kernel initializer | The kernel initializer does not affect the result but sometimes the model can have an explosion of the gradient in the very early training. The Lecun normal kernel fixed that. |
| Weight of the KL loss | The impact of the weight is not significant on the model if it is set up in non-extreme values. The default is 0.5. |
| Reconstructed loss | The best reconstructed loss is MSLE way ahead of MAE, MSE, RMSE. |

*Generation of artificial AGE*

We implemented the same strategy as described in the study of Brechtmann *et al.* (Brechtmann et al., 2018). Briefly, raw read counts in the original dataset were replaced by:

$$k_{ij}^{O}=round(s_{i}2^{\mu_{j}^{u}\pm\exp\left( N \right)\sigma_{j}^{u}})$$

With $k_{ij}^{O}$ the generated count for gene *j* and sample *i* to substitute to the original value, $s_{i}$ is the size factor estimated by DESeq2 (Love et al., 2014), $\mu_{j}^{u}$ the mean of $u_{ij}$ for gene $j$ and $\sigma_{j}^{u}$ the standard deviation, where $u_{ij}=\log_{2} \left( \frac{k_{ij}}{s_{i}}+1 \right)$ and $N$ is the amplitude and  is drawn from a normal distribution characterized by a mean of log(3) and a standard deviation of log(1.6).

**References**

Abadi, M., Agarwal, A., Barham, P., Yu, Y., and Xiaoqiang, Z. (2015). TensorFlow: Large-Scale Machine Learning on Heterogeneous Distributed Systems.

Brechtmann, F., Mertes, C., and Gagneur, J. (2018). OUTRIDER: A Statistical Method for Detecting Aberrantly Expressed Genes in RNA Sequencing Data.

Kingma, D.P., and Welling, M. (2013). Auto-encoding variational bayes. ArXiv Preprint ArXiv:1312.6114.

Love, M.I., Huber, W., and Anders, S. (2014). Moderated estimation of fold change and dispersion for RNA-seq data with DESeq2. Genome Biology *15*, 550.

**Supplementary Figure 1. Divergence score, delta count and anomaly score for the 6 pathogenic candidates in Kremer et al.** A) Pathogenic candidates identified by Kremer and ABEILLE. *TIMMDC1* was identified by AGE and aberrant splicing in two patients MUC1344 and MUC1365 and *MGST1* was identified by AGE in patient MUC1396 in Kremer study and confirmed by ABEILLE. B) Pathogenic candidates identified by Kremer not confirmed by ABEILLE. *MCOLN1* in patient MUC1361 was identified by AGE although with a signal slightly inferior of the thresholds of significativity for Z-score and p-values set by the authors, *ALDH18A1* was identified by aberrant splicing with AGE at the limit of thresholds for patient MUC1404, finally in patient MUC1350, *CLPP* was identified by allele specific-expression only without any consequences on the expression level.

Kremer

ABEILLE

OUTRIDER

**A**

**B**

**C**

A

E

D

C

B

Kremer

ABEILLE

OUTRIDER

F

G

H

I

**Supplementary Figure 2. Functional analysis of AGEs identified by ABEILLE, OUTRIDER and the case-control approach on the Kremer dataset.** Enriched terms as found by EnrichR on AGEs identified by ABEILLE (first column), OUTRIDER (second column), and in the original study (third column). The first 10 significantly enriched terms are showed for A) GO Biological processes, B) GO Cellular components, C) GO Molecular functions, D) Human Phenotype Ontology (HPO), E) Jensen Diseases, F) DisGeNET, G) Rare Diseases AutoRIF, H) Rare Diseases GeneRIF, I) ClinVar. The number of genes is reported on the horizontal axis. Significance expressed as -log10(p-value).

**Supplementary Figure 3. Benchmark of ABEILLE and OUTRIDER on semi-synthetic data.** We run ABEILLE and OUTRIDER on 18 semi-synthetic datasets composed by increasing number of samples, namely 50, 75, 125, 250, 500, 1000 and with different proportion of *in silico* generated AGE as indicated in the legend in the plot. We then calculated the precision-recall curves by sorting detected AGE by p-values and Z-scores for OUTRIDER and by Divergence score and Delta Count for ABEILLE.
